# Herd-Level Modeling of Bovine Viral Diarrhea Virus (BVDV) Transmission in Cattle Herds in Southern Chile: Linking Within and Between-Herd Dynamics

**DOI:** 10.1155/2024/4734277

**Published:** 2024-10-28

**Authors:** Oscar Alocilla-Velásquez, Gustavo Monti, Helmut Saatkamp, Monique Mourits, Ann Lindberg, Stefan Widgren

**Affiliations:** ^1^Department of Veterinary Sciences and Public Health, Natural Resources Faculty, Universidad Católica de Temuco, Temuco, Chile; ^2^Quantitative Veterinary Epidemiology, Wageningen University, Droevendaalsesteeg 1 (Campus) Building No. 107, 6702 PB, Wageningen, Netherlands; ^3^Business Economics Group, Department of Social Sciences, Wageningen University, Hollandseweg 1, 6706 KN, Wageningen, Netherlands; ^4^Department of Disease Control and Epidemiology, Swedish Veterinary Agency, Uppsala 751 89, Sweden

**Keywords:** BVD, BVDV, modeling

## Abstract

Bovine viral diarrhea (BVD) represents a serious threat to the cattle sector in Chile, indicating the need for a regionally defined control program. *Ex-ante* evaluations of program options using simulation modeling have proven to be a successful approach in providing decision-makers with relevant supporting insights in that respect. Given the complexity of bovine viral diarrhea virus (BVDV) infection dynamics, simulation of BVD spread in a metapopulation requires detailed consideration of both within and between herd transmission dynamics. The aims of the study are (i) to investigate the dynamics of BVDV transmission in cattle herds in southern Chile by linking a within-herd transmission model (WHM) that accounts for the BVDV's unique characteristics with a between-herd model (BHM) that meets the demands for further regional control strategy evaluation; (ii) to suggest and discuss criteria for evaluation of the model approach and plausibility for later research and for support decision-making. This resulted in bringing forth a modeling rationale for complex disease spread simulation in metapopulations. BHM simulations under this approach show outcomes that agree with BVDV's known situation in Chile; dairy herds prevalence at endemic equilibrium reaches and maintains 75%, which agrees with estimations of BVDV active infection in dairy herds in southern Chile (77%). For the entire herd population, the infection always reaches endemic levels with a large proportion of infected herds (median = 60%), where herd prevalence was higher in the dairy herd class than in the remaining categories. Transmission probability variation affects the new infections picked, prevalence at endemic levels, and the velocity in which the infection spreads between herds. The fact that the presented approach was able to model a complex infection dynamic such BVDV, with sufficient confidence, provides evidence that this approach can be used to explore mitigation strategies to control BVDV in southern Chilean herds.

## 1. Introduction

Bovine viral diarrhea (BVD) is a ubiquitous endemic disease in most bovine populations [1] caused by a pestivirus named Bovine viral diarrhea virus (BVDV). The infection causes a broad spectrum of clinical manifestations that depend on the physiological status of the host and can vary from reproductive problems (abortion, stillbirths, gestation of persistently infected animals) to mild or unapparent clinical signs [2]. BVD is one of the most critical diseases in cattle in terms of economic impact, leading to high losses due to its reproductive impairment, immunosuppression effect [3], and costs to control it. These losses vary between herds and between countries depending on the status of the disease [4, 5].

Once a susceptible (Sus) host is infected, the disease can manifest in two ways depending on when the infection was acquired. Infected hosts can shed the virus transiently after an acute infection period (transiently infected [TI]) or shed the virus persistently for life (persistently infected [PI]) if they get infected in utero before 150 days of gestation approximately. If the fetus gets infected after 150 days of pregnancy, the immune (Imm) system is competent, and the infection can lead to abortion, stillbirth, congenital disabilities, or the birth of a normal calf [6].

Even though the mortality rate of PI animals in the first year of life is high (over 50%) [7], some PI females can survive until calving, always giving birth to a PI calf. Under natural conditions, direct close contact between a PI and a Sus animal is considered the most effective transmission method [8]. Direct contact between a TI and a Sus animal can also result in an infection but is considered less effective [6]. One crucial feature of BVDV transmission is the presence of non-PI cows carrying a PI fetus (so-called “Trojan Cows”). These dams seem healthy and are Imm to BVDV, but their calves represent a critical risk to the herd due to the large and lifelong amount of virus that they shed [9].

BVDV transmission in a herd depends on the prevalence of infected animals (PI specially), the contact rate between animals, the virulence of the virus strains, and the susceptibility of the hosts [6]. Herd structure and management are, therefore, crucial aspects of BVDV spread dynamics [9, 10].

Essential sources for the introduction, reintroduction, and spread of BVDV between herds are the acquisition of pregnant cows or heifers carrying a PI fetus [11], neighboring contacts between infected and Sus herds [9, 12], the use of contaminated semen [13] and natural services with PI or TI bulls [14].

BVD in Chile is highly prevalent; the true herd prevalence of dairy herds with active infection was estimated at 77%, and the true individual prevalence of dairy cows with active infection was estimated at 3.5% in Los Rios and Los Lagos region, where open herds and herds that mix animal categories are more likely to have higher prevalence [15]. On the other hand, the situation in beef herds is outdated, the latest study (1997) estimated seroprevalence in fattening animals in 86% with 100% of herds with reacting animals [16]. The virus was first isolated in 1986 from a calf with mucosal disease [17], and the genomic study evidences genotypes BVDV-1 with the subtypes 1a,1b,1j, and BVDV-2, being the most frequent isolated BVDV-1j (51.4%) [18], this genetic diversity differs with isolates from South America, Europe, and North America, due to frequency of BVDV-1j and differences in amino acid sequence in BVDV-1b in comparison to isolates from Argentina and Brazil of this isolates [18]. Furthermore, southern Chile is the most important zone for dairy production and cattle farming in the country, accounting for 44.5% of the national cattle population and nearly 80% of the dairy production of the country [19]. Regardless of this background, no control program exists to this date in the country; the efforts to control the disease are nonsystematic herd-level initiatives that are launched when clinical evidence is observed. Hence, BVD poses a serious threat to the cattle sector within a large area, indicating the need for a regionally defined control program. The complexity in the infection dynamics of BVDV however, introduces many uncertainties about the epidemiological (and economic) effects of the various control options (e.g., transport restrictions, vaccination, test, and culling). Understanding the nature of these uncertainties is crucial in selecting the most suitable control option. *Ex-ante* evaluations of program options by means of simulation modeling have proven to be a successful approach in providing decision-makers with relevant supporting insights in that respect [20–22].

*Ex ante* evaluations of this nature have primarily been conducted for highly contagious illnesses like Avian Influenza, foot-and-mouth disease (FMD), and Swine Fever [20, 21, 23]. Nevertheless, significant distinctions emerge when comparing these diseases with BVD. For instance, in the case of the former diseases, in infected farms, a large proportion of animals (infected, Sus at-risk animals) are promptly culled, and the spread within farms is less intricate compared to BVDV infections (PI and TI presence, and trojan cows complicate infection dynamics). Therefore, modeling these diseases at a metapopulation level can be a more straightforward task, in contrast to addressing BVDV, due to complex within-herd dynamics.

Given the biological and epidemiological complexity of BVDV infection dynamics, to realistically simulate the spread of the virus in a metapopulation, it is necessary to consider both—within and between transmission dynamics in detail. The inclusion of fundamental features such as the likelihood of the occurrence of TI, PI, and Trojan cows, the role of herd immunity in the acquisition or clearance of an infection process, and the risk of spread to other herds are required, which makes modeling BVDV between herds difficult and computationally intensive. Nevertheless, a few BVDV between herd transmission models have been developed so far [24–27], using different approaches such as coupling within and between dynamics and network modeling with less consideration of intraherd characteristics. Given the lack of actual data in Chile, modeling BVDV transmission between cattle herds, considering local characteristics, can be a powerful tool to evaluate the efficiency and economic impact of future possible mitigation strategies programs. However, the availability of this type of framework for their application to other population realities and the subsequent generation of information for decision-making is difficult. Therefore, the development of a different approach that allows the inclusion of relevant within-farm and between-farm characteristics, not only for BVD but also for other infectious diseases, where pathogen spread is moderate, and problems such as complete culling are not an option for controlling it, could be a useful tool for dealing with alternative control programs.

This study aimed to (i) investigate the dynamics of BVDV transmission in cattle herds in southern Chile by linking a within-herd transmission model (WHM) that accounts for the unique characteristics of the BVDV infection dynamic with a between-herd model (BHM) that meets the requirements for a regional control strategy; (ii) to suggest and discuss criteria for evaluation of the model approach and plausibility for later research and for support decision-making.

## 2. Model Description

### 2.1. Target Population

**Figure 1:**
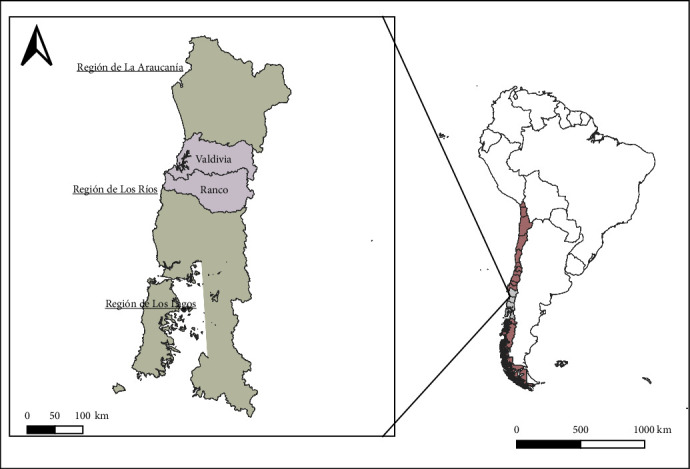
Study area: geographical location of Los Rios, with their two provinces (Valdivia and Ranco), La Araucanía and Los Lagos regions, Chile.

This study focuses on cattle herds in southern Chile, specifically in the Los Rios region, which consists of two provinces: Valdivia Province and Ranco Province (Figure 1). The Los Ríos region is one of the most important regions for Chilean livestock, accounting for 20% of the national cattle mass and responsible for 30% of the country's milk production. It also has 15% of the beef cattle population [19]; and, as mentioned above, BVDV is highly prevalent in this zone [15].

### 2.2. Rationale of the Modeling Approach

A WHM was coupled with a BHM to study the unique dynamic of BVDV transmission in cattle herds. Various software options were explored to find those that captured the necessary features to model BVDV transmission between herds. Interspread Plus (ISP) V6 [28] was chosen, since it has been used extensively to model transmission of highly contagious diseases [20–22, 29] and for containing most of the needed features. However, the use of ISP was complemented by the SimInf R package [30, 31], as the within-herd transmission capabilities of ISP were limited for including all major BVDV transmission pathways. This combination of packages allowed the coupling of within-herd dynamics to fully model the transmission of BVDV between herds. A general overview of the framework and modeling approach used is described in this section.

#### 2.2.1. BHM by ISP

ISP is a framework for modeling between-herds pathogens transmission; it is described in detail in [28], and therefore, only a brief overview is provided here. Using a spatially explicit and stochastic approach, ISP can simulate the spread of pathogens between infected and Sus farms through animal movement, local spread, and airborne spread. Control strategies, including measures such as surveillance, culling, vaccination, and movement restrictions, can also be configured to assess their impact on transmission. All spread and control mechanisms operate spatially by using the geographical location of farms and contain variation and uncertainty (mostly reflected by empirical probability functions) using Monte Carlo simulation. Multiple replications, each representing a possible course of an epidemic, are run to gain insight into the possible range of outcomes. One limitation to the use of ISP, is the lack of a module that allows us to simulate pathogen spread within a herd in a detailed and complex manner as needed for the objectives of this study. The within-herd characteristics are basically represented in the parameterization of latent and infectious periods, and the time from infection to the appearance of clinical signs after a herd has been infected. This is clearly not enough for BVDV within-herd dynamics. This is important for BVDV because within-herd dynamics is related to herd immunity/susceptibility and how this relates to the risk of spread to other herds. Therefore, ISP is limited in its ability to fully model BVDV dynamics, and a complement to overcome the limitations is needed to make ISP-modeling workable.

#### 2.2.2. WHM by SimInf

The R package SimInf is characterized by its flexibility and efficiency in handling within-herd and between-herd dynamics in the form of network data, and it is designed for data-driven spatio-temporal disease modeling [31]. SimInf treats infection dynamics as continuous-time Markov chains using the Gillespie stochastic simulation algorithm. Demographic data or animal movements can be incorporated as scheduled events and used to change the state of a group within the population at a predefined time, e.g., group vaccination, external movements, and exits. However, as SimInf is designed to perform data-driven disease spread simulation, a complete ex-post database at the individual and herd level is required to fully exploit the capabilities of SimInf.

Due to the lack of high-resolution animal movement data, we restricted the use of SimInf in this study to the within-herd dynamics only. Given the previously indicated limitations of the frameworks used, a two-step process was developed to link within and between-herd BVDV dynamics models for southern Chile conditions. The process consists of using the within-herd simulation in SimInf to obtain a set of profiles that are sufficiently representatives of the most likely scenarios following a herd infection but at the same time limited enough to make the between-herd modeling still feasible once these profiles are used as input in ISP, as explained in more detail in the following sections.

### 2.3. WHM

**Figure 2:**
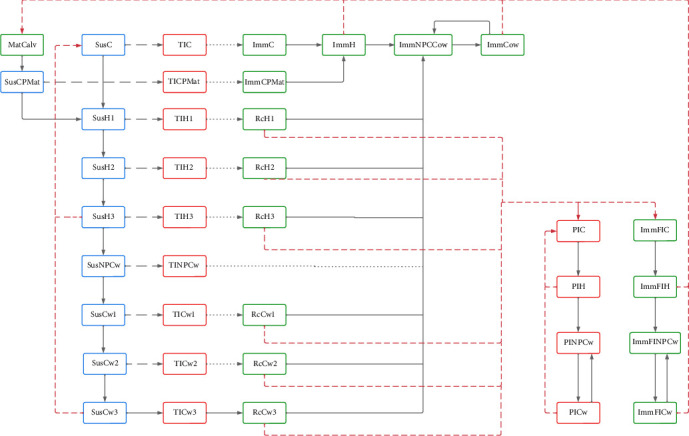
The figure describes the within-herd dynamics for a dairy herd. The herd has been divided into four groups: Calves (C), heifers (H), cows (Cw), and nonpregnant cows (NPCw). In addition, H and Cw were further divided into three age categories to correspond to the gestation trimester (H1, H2, H3, Cw1, Cw2, Cw3), with the purpose to address infection results regarding pregnancy stage (e.g., an earlier infection increases the probability to obtain a PI). Males were removed at birth, and culling was possible in each age group at a culling rate of 0.2 per year. At 400 days of age, calves (C) move to H1 (representing the age for a first pregnancy) [32]; then, heifers move every 93 days, first to H2 and then to H3. After calving, H3 moves to NPCw, representing the time between calving and pregnancy (60 days) [33]; after this period, the animal moves to C1 and then every 93 days first to C2 and then to C3 stage for calving and then back to NPCw again. The first step was to run the model without the introduction of infected animals, with the sole purpose of evaluating the herd dynamics in relation to age, production conditions, and management. The population was classified into several mutually exclusive health states with respect to BVDV infection: Susceptible (Sus), transiently infected (TI), persistently infected (PI), immune (Imm), recovered (Rc), immune fatally infected (ImmFI), maternal immune calves (MatCalv).

A WHM for dairy, mixed, and beef herds was built in SimInf. The outcome of these models represented the internal infection dynamics in ISP. Simulations were performed for dairy, mixed, cow-calf, and fattening beef herds of small (<100 animals), medium (101–300 animals), and large (>300 animals) sizes. Infection parameters were taken from the literature, and the population dynamic was designed to resemble a typical Chilean herd. The simulations were run for 12 years, and 500 iterations were evaluated for each model to capture the variability in the results. Except for the fattening herds for which a shorter simulation period (2 years) was used, based on the average time that fattening cattle are reared in Los Rios and Los Lagos region, up to 480–500 kg before slaughter (Figure 2).

#### 2.3.1. Within-Dairy Herd Dynamics

PI and TI animals are responsible for spreading the virus at a rate *β*_P_ and *β*_T_, respectively; transmission between age groups is only possible through PI animals at a rate *β*_bg_; model parameters are presented in Table 1. The infection's outcome depends on the Imm status and the age category of the animals.

If a SusC is infected, a 14-day transient infection period begins (Table 1), after which lifelong immunity is assumed (as for the remaining time as a calf, heifer, and adult cow), and she will give birth to calves protected for 180 days by maternal antibodies. If a pregnant Sus heifer (SusH1, SusH2, SusH3) or cow (SusCw1, SusCw2, SusCw3) gets infected, a 14-day transient infection was handled (TIH1, TIH2, TIH3, TICw1, TICw2, TICw3, TINPCw). However, the consequences for the calf varied depending on the trimester of pregnancy in which the dam was infected [39]. The states RcH1, RcH2, RcH3, RcCw1, Rcw2, Rcw3 represent the trimester of pregnancy in which the animals recovered (Rc) after a transient infection and also define the possible consequences of pregnancy infection. The possible consequences of pregnancy infection and their probabilities are detailed in Table 2.

The model assumes that calves with congenital defects are culled after birth and, therefore, do not pose a risk of spreading the virus to other animals. Fetal-infected calves (FIC) will remain Imm for life, and female calves will give birth to calves protected by maternal antibodies (ImmMatC). If a PI calf is born, it will remain PI for life, with a short life expectancy (Table 1); however, if a female PI calf grows up and becomes pregnant, she will always give birth to PI calves. Equation (1) gives the transition rate from Sus to infected animals (TI):

(1) $$\begin{array}{c} Sus\to TI_{\left(g,t\right)}=Sus_{\left(g,t\right)}\left[\begin{array}{c} \beta_{g}^{P}\times PI_{\left(g,t\right)}+\beta_{g}^{T}\times TI_{\left(g,t\right)}+\sum_{b\neq g}\beta_{b}^{P}\times PI^{b} \end{array}\right], \end{array}$$

where *β*_**g**_^**P**^ and *β*_**g**_^**T**^ correspond to the daily transmission rate for PI and TI in each group (**g**) at time (*t*), **PI**_(**g**, **t**)_ and **TI**_(**g**, **t**)_*I*, correspond to the number PI and TI animals in each group (**g**) at time (*t*), *β*_**b**_^**P**^ correspond to daily transmission rate between groups within a herd due to **PI**^**b**^ (PI animals from groups *b*) (Table 1), in the model TI transmission was assumed to be only within groups, meanwhile PI transmission was assumed to be within and between groups, the transmission is assumed to be density dependent. The default scenario corresponds to the introduction of a PI heifer in a fully Sus population.

#### 2.3.2. Within-Mixed and Beef Herd Dynamics

The fattening process is characterized by the purchase of male calves from dairy farms and dual-purpose steers from mixed or other herds. For the sake of simplicity, a standard fattening system was simulated to capture the most common practices. Most herds purchase weaned male calves, which are fattened to a weight of ~480–500 kg before slaughter or sale. Mutually exclusive age groups represent eight age categories that corresponding to the trimonthly path from purchase to the end of the fattening process. For, animals of different ages are assumed to enter at the same time at the start of the simulation.

For the fattening herds, it was assumed that the purchased steers arrived without BVDV immunity and that a PI steer arrived with them. Sus, TI, PI, Imm, Rc are the possible health status of the animals in all age groups. As for dairy herds, the source of infection is TI and PI animals; transient animals recover at a rate of *Rcr*, culling or deats is possible at any time, and after 24 months, all animals are removed from the system (sold or transferred to finishing farms). The following equation gives the transition rate from Sus to infected animals in fattening herds.

(2) $$\begin{array}{c} Sus\to {TI}_{\left(g,t\right)}={Sus}_{\left(g,t\right)}\left[\begin{array}{c} \beta_{g}^{P}\times {PI}_{\left(g,t\right)}+\beta_{g}^{T}\times {TI}_{\left(g,t\right)} \end{array}\right]. \end{array}$$

For mixed herds, the within-population dynamics were assumed to be the same as for dairy herds, the only difference being that male calves are kept in the herd until weaning. Cow-calf herds are assumed to have the same dynamics as dairy herds. Therefore, the same transition probabilities were used. Infection parameters such as TI and PI transmission rate and recovery rate were the same as those used for dairy herds WHM.

##### 2.3.2.1. Overall WHM Evaluation

Once the population dynamic had become dynamically stable in the absence of infection, this equilibrium point for the population distribution was used as the initial condition for the simulations in which the infection was introduced (by a PI-infected pregnant heifer). As the aim of the WHM simulation was to obtain a set of inputs for a between-herd transmission model (BHM) and not to assess in detail the within-herd transmission, the evaluation of the WHM results was essentially to assess the overall consistency with the existing literature, to increase the credibility of the transition matrix and its probabilities. Therefore, the behavior of the model was assessed by evaluation of virus persistence probability (PP) (probability that the infections persist in time) by plotting Kaplan–Meier curves and comparing them with existing literature. Only the WHM dairy herds were evaluated in depth because the majority of BVD models in the literature focus on dairy herds, which facilitates comparison and validation, and because dairy herds have more complex BVDV dynamics in comparison to beef herds. No reintroduction was allowed for ant built WHM.

##### 2.3.2.2. Defining and Obtaining Herd-Profiles for BHM Input

As a result of the study of WHM results and behavior, six herd profiles were defined to resemble the within-herd infection dynamics in the BHM. Herd-profile definition criteria were based on (i) herd status (infected or cleared); (ii) herd immunity, defined by the proportion of Rc cattle after a 14-day infection period [41], related with the infection persistence post virus introduction; (iii) ISP features. Therefore, herd profiles compose a set of mutually exclusive compartments, which can be introduced in ISP as a “farm state.” Herd-profiles were defined as follows:

• Infected and low immunity (ILI): Herds with at least one PI or TI or Trojan cow present and a proportion of Rc animals up to 25%.
• Infected and medium immunity (IMI): Herds with at least one PI or TI or Trojan cow and a proportion of Rc animals ranging between 25% and 75%.
• Infected and high immunity (IHI): Herds with at least one PI or TI or Trojan cow and a proportion of Rc animals greater than 75%.
• Clear and low immunity (CLI): Herds with no PI, TI, or Trojan animals present and a proportion of Rc animals up to 25%.
• Clear and medium immunity (CMI): Herd with no PI, TI, or Trojan animals present and a proportion of Rc animals ranging between 25% and 75%.
• Clear and high immunity (CHI): Herds with no PI, TI, or Trojan animals present and a proportion of Rc animals of more than 75%.

We do not consider a fully Sus postinfection stage given the high herd prevalence in Chile [15, 42] and the long-time span required to reach this state. In the absence of reinfection, the time to become fully Sus again will depend mainly on the replacement rate.

The final herd state for each time step was classified into one of the previous herd-profiles, thus constructing a transition matrix and obtaining a transition probability between herd-profiles (Tables 3–5). The simulations were run with a time step of 90-days; this interval was chosen because:

• The information obtained on new infections, PI presence, and Rc animals was sufficient when compared to 60- and 30-day time steps. It corresponds to the trimesters of the gestation period, which facilitate the simulation of BVDV infections in pregnant cows.
• A 90-day time step is sufficiently informative enough and helps to save computational resources, especially for long-term scenarios where we want to evaluate significant changes at the herd level (e.g., changes in herd profile) and the evolution of the infection in a metapopulation.
• Reducing this time step (e.g., to 30 days/1 month) would increase the accuracy of the result, but the associated increase in computational time and resources is not justified. Therefore, 90 days was a good compromise between accuracy and time.

#### 2.3.3. BVDV Between-Herd Transmission Model

**Figure 3:**
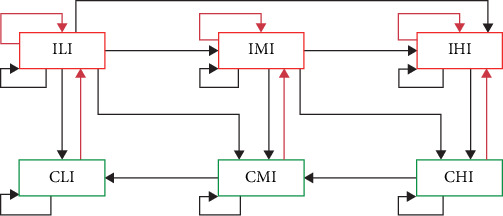
Simulated herd-profiles pathway of BVDV infections. Red boxes represent all infected herd-profiles, and green boxes represent all cleared herd-profiles. CHI, clear high immunity; CLI, clear low immunity; CMI, clear medium immunity; IHI, infected high immunity; ILI, infected low immunity; IMI, infected medium immunity.

The herd profiles obtained in the previous step were introduced into ISP-BHM as mutually exclusive compartments, alongside transition probabilities between them. The conceptualization of the BHM model using the herd-profiles is shown in Figure 3. The black arrows represent the trajectory that a herd can follow after virus introduction at each 90-day time step. The red arrows represent the result of effective transmission by cattle movement. For example, if a herd in the CLI stage becomes infected due to a successful introduction, it will become ILI (infected but with the same low immunity level). In the next time step, three transition outcomes are possible: remaining at the same stage (ILI), moving to a higher immunity level (IMI or IHI), or clearing the infection with the same immunity level (CLI).

To take into account transport-specific characteristics related to the type of livestock activity, the BHM distinguishes seven herd classes:

• Dairy herds: Herds whose main activity is the sale of milk production.
• Cow-calf herds: Herds whose main activity is the rearing of beef cattle.
• Fattening herds: Herds whose main business is fattening animals from dairy, mixed, cow-calf, or other herds.
• Mixed herds: Herds whose main business is milk production and breeding or fattening.
• Other large herds: Herds whose main business is not milk or beef production, which keeps cattle for various purposes.
• Markets: Live cattle auctions.
• Sport exhibition: Exhibition centers and rodeo grounds.

To select the herd class in which infection would start for the simulation, one round of simulation was run for each herd class and size to assess whether model results and behavior were strongly dependent on the starting herd class. The virus was introduced into a randomly selected herd for each herd class and size; these simulations were run for 30-time steps (2700 days) and iterated 100 times. One of the previous simulations with more variability was then selected and run for 50-time steps and 200 iterations for further detailed analysis of model behavior and results.

##### 2.3.3.1. Between-Herd Transmission Setting in ISP

A farm file containing details of the population at risk is the starting point for ISP modeling; this file contains a unique identifier, location details (cartesian coordinates), farm class, and animal number. The farm classes are given in Table 6 and are defined based on location (provinces of the Los Rios region), type, and size of the herds.

The herd profiles and their transition probabilities were obtained as described in the previous section, and they were specified in ISP in the “set state” section for each herd type (dairy, mixed, cow-calf, beef). Thus, in every time step, the ISP reads the “set state” section, and the herds change their state according to the probabilities specified as proportions in the named section. Shipment details and the probability of transmission are specified in the “Movement Type” section of the ISP. Cattle movements data from 3.800 herds in the Los Rios region [43] were used to obtain the parameters required for the BHM (i.e., animal movement frequency and destination probability parameters). This data included movements between and within the two provinces that form the Los Rios Region (Ranco and Valdivia), excluding any movement interactions with herds from neighboring regions. Movement frequency was introduced by setting the 90-day average number of shipments as the lambda parameter for a Poisson distribution (Table 6). The destination probability (Supporting Information 1 and 2) for every herd category was estimated as the proportion of shipments to a given location from a given herd category and introduced as a constant value by estimating the proportion of shipments to a given location from a given herd category assuming that this probability did not change during the simulation. The infection started in a randomly selected herd from the “other-medium herd” class; the model considers the transmission through animal movement as the only transmission route, neighboring infection likelihood was not considered due to the absence of this information for the country, and it was considered less likely do to the predominant extensive productive system in the country. In the absence of information on between-herd transmission parameters, the transmission probability by shipment (TP) was estimated for large, medium, and small herd sizes as a function of the average number of animals introduced in each shipment and the within-herd prevalence, obtaining the probability of introducing at least one infected animal (Table 7), and later set in the model as a constant value. Since individual prevalence values in Chile are only updated for dairy herds [15], those reported values for within-herd active infection prevalence were used for all herd classes. The BHM allows for the reintroduction of BVDV throughout the simulation period.

The probability of introducing at least one infected animal was calculated using the cumulative probability function (pbinom) from R software [30], using the following equation:

  $$\begin{array}{c} TP=1-pbinom\left(0,n,p\right), \end{array}$$

where *n* = average number of animals in every shipment; *p* = within-herd prevalence of infected animals for a given herd class.

#### 2.3.4. BHM Outcomes

The aim of the BHM is to provide an analytical framework for assessing the impact of mitigation strategies on herd prevalence. The results of the evaluation were therefore used to validate the model. The validation (credibility check) was based on a comparison with the current situation of BVDV herd prevalence in southern Chile. The persistence of the infection by herd type and the average number of infected herds over time by herd type were also analyzed. A sensitivity analysis was performed by modification of the probability of transmission probability by shipments (TP) in the BHM, given the uncertainty in animal level-prevalence for herd classes besides dairy herds. For this purpose, the animal level prevalence “*p*” from Table 1 was modified by half and double of the original value.

## 3. Approach Evaluation and Results

### 3.1. Main Features of BVDV-WHM Using SimInf

The results of infection simulations for small, medium, and large herds, were very similar, therefore, for simplicity, we show results only for a medium dairy herd.

**Figure 4:**
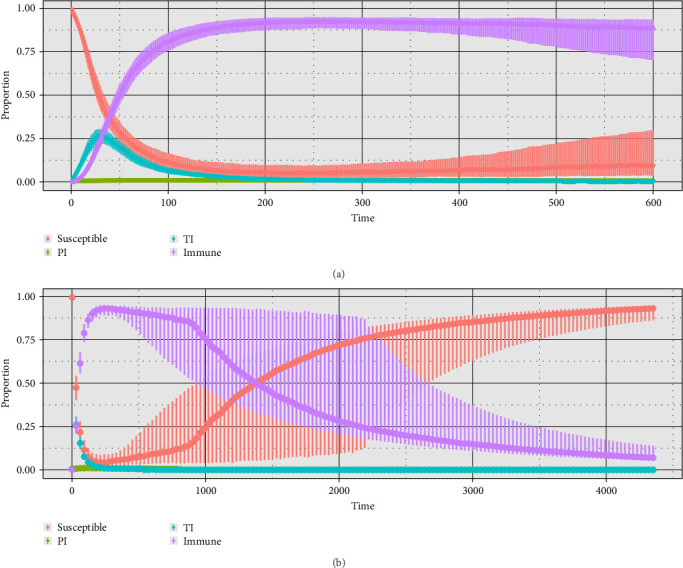
BVDV outbreaks simulation results for a medium fully Sus dairy herd after a PI heifer introduction, time is shown in days. (A) The figure shows the first 600 days of the outbreak with median and interquartile range. (B) The figure shows the entire 12-year period with median and interquartile range. BVDV, bovine viral diarrhea virus; PI, persistently infected; Sus, susceptible; TI, transiently infected.

**Figure 5:**
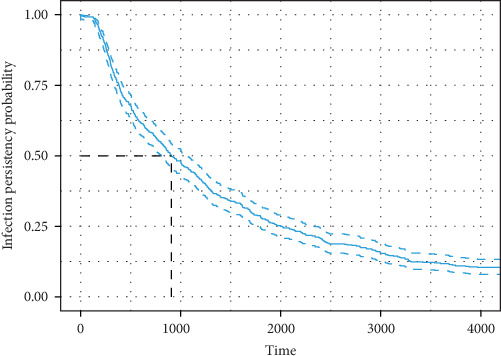
Infection persistency probability of BVDV, after a PI heifer introduction in a medium dairy herd (95% CI). BVDV, bovine viral diarrhea virus; CI, confidence interval; PI, persistently infected.

After a short epidemic period (Figure 4A), the model reached an equilibrium before 600 days, where ~30% of the animals in the herd are infected. Thereafter, the proportion of TI infections decreases, and the proportion of Imm animals increases until 80% of the animals are protected around day 150, with the proportion of PI animals remaining low throughout the period. Figure 4B shows the results for the 12 years of the simulation. At day 1500 (4 years), the median proportion of Sus animals starts to increase significantly, reaching an equilibrium at around 4000 days (11 years), where over 80% of the animals are Sus again. In 90% of the 500 iterations performed, clearance was observed throughout the simulation period.  Figure 5 shows the median PP, closely to day 900; the PP is 50%, decreasing in time to become nearly 12% by the end of the simulation.

### 3.2. Outcome of BVD-Herd-Profiles

Tables 3–5 show the transition probabilities between herd profiles for small, medium, and large dairy herds resulting from the WHM study and that will be used as input in the ISP. The transition probabilities between herd profiles for fattening and mixed herds are shown in Supporting Information 3.

### 3.3. Inclusion of BVDV-Profiles in Between Herd Modeling in ISP

**Figure 6:**
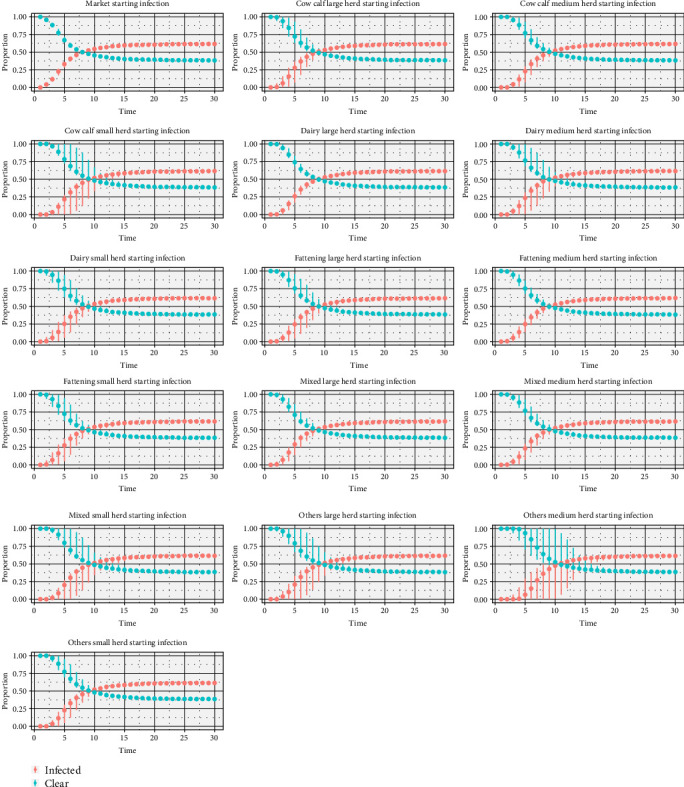
Starting situations of BVDV infection in the metapopulation, median, minimum, and maximum proportion of infected and cleared herds in the metapopulation. BVDV, bovine viral diarrhea virus.

The simulation results for the different herd classes as the starting point of infection were first evaluated (Figure 6). In most simulations, 50% of the herds become infected between time steps 5 and 10 (450–900 days), reach similar endemic levels around time step 20 (1800 days), and remain there until the end of the simulations. A striking feature is that the variability of the results also varies considerably between the different starting situations. For example, when the infection starts in markets, dairy, or mixed large herds, the variability is extremely low. In contrast, when the infection starts in “others medium herds,” the variability is higher.

**Figure 7:**
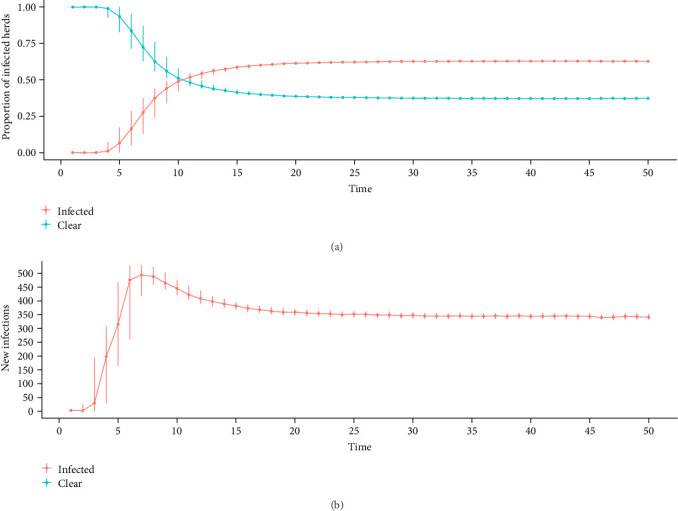
(A) Cumulative proportion median and quantiles of infected and cleared herds and (B) number of new infections in the metapopulation, reinfection allowed for the entire period, the infection starts in an “Others Medium” herd class.

On the basis of higher variability criteria, the model in which the infection starts is in a “other medium” herd class for more detailed analysis (Figure 7A). Before 2.5 years (10-time steps), half of the herds were infected, and the maximum number of infected herds was 2388 at time step 42 (10 years) during the endemic state; this endemic state was reached at timestep 26 (6 years) was 63% of the herds are infected, and this prevalence is maintained until the end of the simulation. Figure 7B shows the number of new infections throughout the simulation; the peak of the outbreak is reached before time step 10 (2.5 years), and after time step 25 (6 years), the number of new infections remains constant with low variability when the endemic state is reached. Over the 200 iterations, no self-extinction was observed, and in each iteration, a significant outbreak was observed, which later reached the endemic state. When the simulations were run for a longer period or for a shorter period with more iterations, no self-extinction was observed (data not shown).

**Figure 8:**
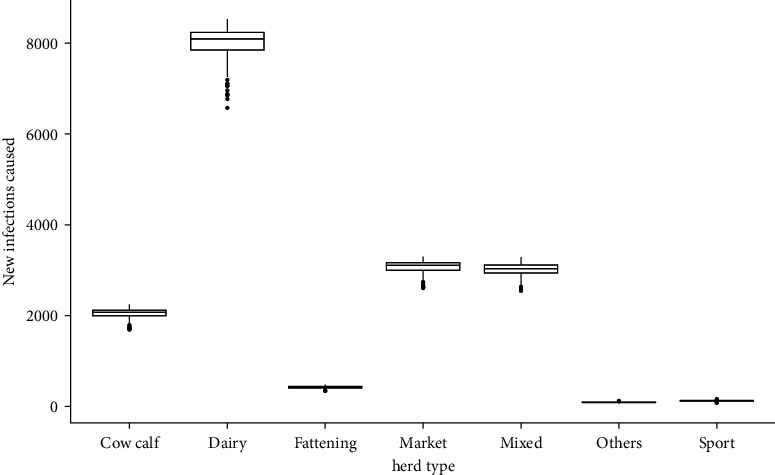
Distribution of new infected herds by herd's types.

Newly infected herds were mainly caused by effective contact with dairy herds, followed by markets and mixed herds; newly infected herds caused by “sports/exhibition centers and other herds” are significantly less important compared to the other herd classes (Figure 8 and Table 8), but may be crucial in later stages of possible control and eradication programs.

**Figure 9:**
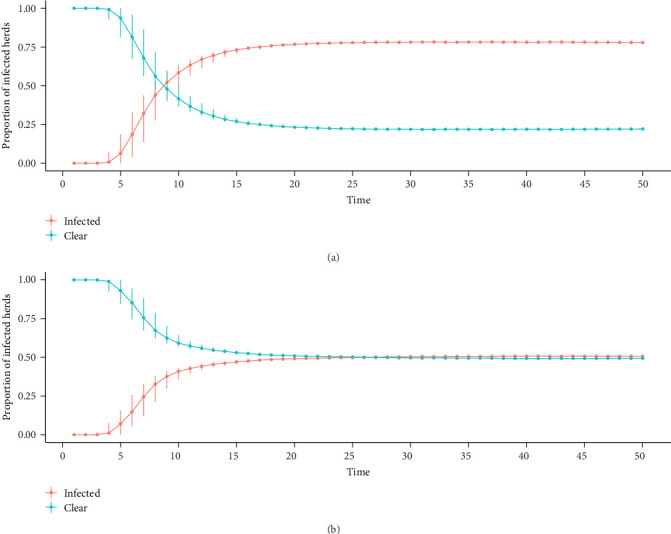
(A) Cumulative proportion, median, and quantiles for dairy and mixed infected and cleared herd classes. (B) Cumulative proportion, median, and quantiles for beef and others infected and cleared herd classes.

When the model results are disaggregated by herd class (Figure 9), it is observed that for dairy and mixed herds, the proportion of infected herds reaches its peak almost at 5 years (20-time steps) and maintains this proportion (75%) for the rest of the simulation, whereas for beef and other herd class, the outbreak reaches its peak around the same time as dairy and mixed herds, but the proportion of infected herds at the endemic state is lower (almost 50%) and maintains this level for the rest of the time span.

### 3.4. Sensitivity Analysis

**Figure 10:**
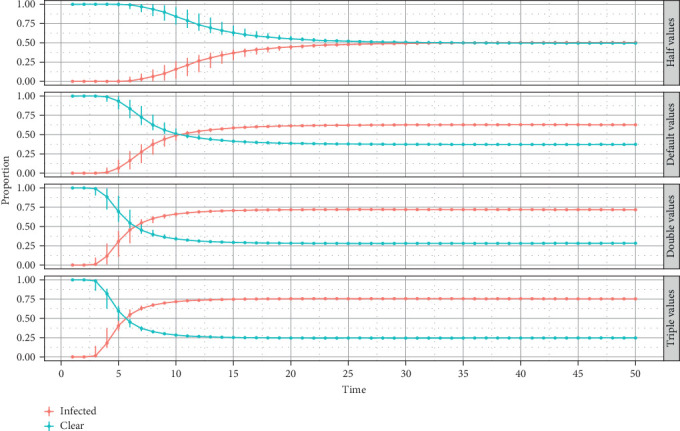
Cumulative proportion median and quantiles of infected and cleared herds based on different sets of transmission probabilities by modifying animal level-prevalence in half, double, and triple the value.

Regardless of the variation in transmission probability values (by modification of animal level-prevalence in the BHM), the infection dynamics always reach the endemic state with a high proportion of infected herds. The infection spreads fast, and the proportion of infected herds is larger when the transmission probabilities increase by two and three times the default value; but the proportion of herds does not exceed 75% for the largest sets of transmission probabilities. In contrast, reducing the transmission probabilities by half compared to the default values causes the infection to spread slowly with a small proportion of infected herds (no more than 50% of infected herds when the endemic state is reached) (Figure 10).

**Figure 11:**
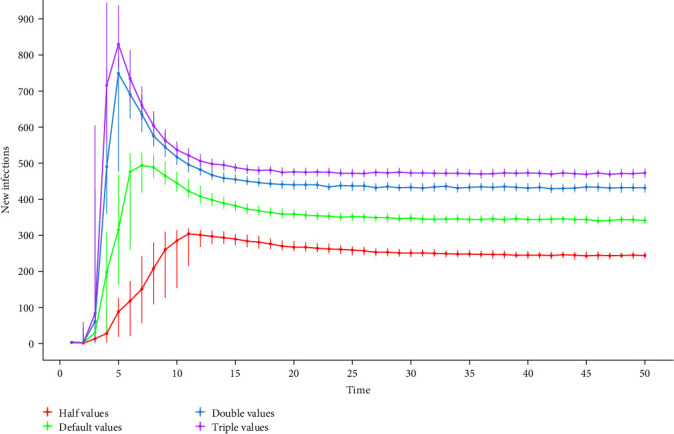
Median and quantiles of the number of newly infected herds based on different sets of transmission probabilities by modifying animal level-prevalence in half, double, and triple the value.

A similar effect is observed for the number of new infections (Figure 11); when the transmission probabilities are set to twice and three times the default values, the outbreak peak is higher and reached earlier, with a constant high number of new infections once the endemic state is reached. On the other hand, reducing the transmission probability by half resulted in a slower infection peak with the lowest number of new infections for the endemic period.

## 4. Discussion

The objectives of this study were (i) to investigate the dynamics of BVDV transmission in cattle herds in southern Chile by linking a WHM that gathers the unique characteristics of BVDV infection dynamic with a BHM that meets the requirements for regional control strategies; (ii) propose and discuss criteria for evaluating model approach and plausibility for further research and decision support. Resulting in bringing forth a modeling rationale for complex disease spread simulation in metapopulations.

### 4.1. Problem Evaluation and Solution Approach

Complex within-herd diseases such as BVD are difficult to simulate at the regional/metapopulation level; therefore, the integration of two useful and validated simulation frameworks, such as ISP and SimInf, was used to overcome this issue.

The current development of ISP modeling has a lot of useful features for between-herd modeling, such as detailed animal movement setting and control strategies, but it does not allow the inclusion of complex within and between-herd transmission dynamics needed for pathogens like BVDV. Therefore, region-wide *ex ante* simulation is not possible in a correct manner. To overcome this, we propose a different method by using a WHM to produce a set of BVDV herd profiles to be used as input in BHM/ISP, as possibilities for herd status taking into account BVDV's unique characteristics, the obtained BHM model meets the requirements for further regional control strategy evaluation.

The approach described must meet several conditions to be justifiable, i.e., it should be consistent with within and between-herd epidemiology (both conceptually and quantitatively). In the case of BVDV, the description of within-herd infection dynamics is fundamental and should, therefore, be valid and justified.

### 4.2. Within Herd Simulation of BVDV

SimInf is a flexible framework for modeling infectious diseases. Its features include ease of customization for new models and characteristics, a set of utilities for inspecting results and postprocessing data, and high performance when simulating an outcome. It has been widely used for disease modeling [44–46] and for BVD simulation as well [27]. Extensive modeling regarding BVD within herd dynamics has been published [35, 36, 38, 47]; hence, considerations for comparison and model validation were available.

Biological and epidemiological crucial aspects of BVD dynamics were contemplated in the WHM to ensure a rational and justified set of herd-profiles for posterior use. The model was built considering several features that impact BVD dynamics within a herd; these features were the age structure of herds, the presence of PI, TI, and trojan cows, the effect of maternal protection, pregnancy infection outcome, infection outcomes (mortality, long-lasting immunity), herd indexes for average Chilean herds (culling rates, calves mortality rates, age of first calving, among others).

### 4.3. Choices of BVDV-Profiles

In our approach, BVDV herd-profiles are crucial; two main characteristics were required for their definition and use: (i) to be representative enough of BVD within herd dynamics and (ii) feasibility for being used as inputs in ISP.

The presence of infected animals (TI or PI) at any given time and the proportion of Rc animals were the used criteria for the definition of herd-profiles, allowing to take cover of two fundamental processes in BVDV infectious dynamics that need to be addressed in the BHM: the risk of introduction into other herds, and the establishment and maintenance of infection within a herd. This was possible due to a SimInf feature that allows to obtain a data frame with the numbers of individuals in each compartment at every time of the simulation, permitting the classification of a herd into one of the defined herd-profiles at any given time. Therefore, in ISP, after a successful virus introduction, the immunity level will define the stage/state (herd profile) for the next time step according to the transition probabilities obtained in the WHM (Tables 3–5 and Supporting Information 3).

By using herd-profiles, we were able to provide a set of exclusive compartments and transition probabilities into ISP to represent within herd dynamics instead of fully modeling a WHM and BHM at the same time, which was not possible in ISP for the case of complex diseases such as BVD. On the other hand, this approach can lack of resolution if changes at the individual level are needed to be studied, for example, PI presence in a metapopulation when evaluating mitigation strategies.

### 4.4. ISP-Simulation

The BHM in ISP allows to explore the BVD dynamics at the regional level, and it will be further used to explore mitigation strategies for decision-making. Outcomes of the model that are useful for this purpose are the proportion and number of infected and noninfected herds according to the different herd immunity levels, the number of newly infected herds, and the identification of the most important infection sources; all this outcome can be further disaggregated by herd characteristics (size, type, location).

### 4.5. Data, Sensitivity Analysis, and Overall Evaluation of the Approach

Here, use Garner and Hamilton in making credible that your approach (including the final outcomes) is useful in regional decision support, specifically aimed at allocating scarce (public and private) resources in the most optimal way

According to Garner and Hamilton [48], four aspects should be addressed to ensure a model reliability: (i) data validity, (ii) conceptual validity, (iii) internal model verification, and (iv) operational validity. Following these guidelines, a step-by-step list of key criteria/components will be pointed out in this discussion.

#### 4.5.1. Coherent Infection and Population Dynamic Representation WHM

Since the first step for the described approach was to obtain a rational and justified set of herd profiles (and their transition probabilities) representing the dynamics of BVDV at the intraherd level, it is necessary that WHM includes critical aspects of BVD epidemiology and biology. Hence, detailed model construction was performed considering all aspects named in Section 4.2 to ensure representativity of within-herd dynamics of the infection.

#### 4.5.2. Overall Agreement Between WHM Outcomes With Field Observations or With Other Simulation Models

The overall agreement between the WHM built-in SimInf and the WHM's available in the literature was evaluated to ensure consistency in the obtained herd-profiles and transition probabilities. The presented WHM is characterized by a short epidemic period in which the proportion of Sus animals declines very rapidly, and the proportion of Imm animals also rises rapidly, accompanied by a small proportion of transient and persistent infection after the epidemic period (Figure 4), similar behavior to the BVDV model behavior was reported by Cherry, Reeves, and Smith [34], the overall PP and the median PP are also consistent with what has been described in the literature [35]. Self-clearance events have been regularly observed in small, medium, and large size herds; these events are related with any factor that influences PI prevalence, the proportion of Sus animals in early pregnancy, and the contact degree among these groups [1]. Therefore, in this model, where no reintroduction of infected animals was allowed, self-clearance events were expected.

#### 4.5.3. Conceptual Validity of the BHM Model

For the BHM, this approach required that the input data be reliable and the used software to be flexible enough to represent the infection dynamics using the profiles obtained from the WHM. Although we were able to represent the path after the introduction of an infected animal into a herd with SimInf, ISP was not designed for such features, e.g., transitions between the six herd profiles in ISP were defined with deterministic probability parameters, as no distributions can be introduced in the “set state” section. Another critical aspect to consider was the probability of movement transmission; no data were available for between-herds transmission rates or probabilities; existing models use approximations based on the transmission rates of PI animals and probabilities of PI introduction of [24, 26]. In view of this, we use the known prevalence of BVDV in Chile (without differentiating between transient and PI animals) and the shipment frequency to estimate the probability of introducing at least one infected animal.

Different initial situations were evaluated as a first approach to examine the behavior of the model, all of them were able to generate an outbreak and reach an endemic state. Also, extremely low variability was observed in the simulations where the infection starts in markets, dairy, or mixed large herds, presumably because those categories show a high animal shipment frequency and to several destinations; therefore, the infection is more likely to spread rapidly; hence less variability is observed at the beginning of the outbreak.

The rapid spread and persistence of the infection in the simulation, regardless on the type of herd in which the infection starts, is influenced by low self-clearance probability, culling rates that are unable to eradicate the infection, high transmission probability driven by the contact structure of the herds and the mere definition of infected herds. This high persistence characteristic is consistent with most reports on the global BVD situation and with simulation models [24, 26].

In the presence of constant animal movement, we were not able to capture iterations (up to 400) in which the metapopulation clears from the infection. This phenomenon is presumably a consequence of the high probability of transmission, the large amount of animal movement, and the reintroduction of infected animals. These results are in agreement with Courcoul and Ezanno [24], who reported that even with a low number of animal movements and without reintroduction, the infection is highly persistent over time.

The number of new infections and the persistence of infection in the metapopulation were shown to be influenced mainly by the transmission probability (Figure 11), with increasing transmission probability leading to a high number of new infections and a high proportion of infected herds when the endemic state is reached. The contact structure of the metapopulation is also involved; in Chile, the contact structure of cattle herds is characterized by being a highly interconnected network, highlighting the role of markets as central nodes, making it easier for a pathogen to spread and likely to cause a large outbreak. Most infections occur in fattening herds, with dairy herds being the main source of infection (Figure 8, Table 8), followed by markets and mixed herds. This is because of the higher risk of introduction due to the frequent purchase of young male calves from dairy herds, which are the main source of trade for cattle herds, especially fattening herds [49], which are usually dead ends for trade.

#### 4.5.4. BHM Outcome Agreement With BVD Chilean Situation

A recent study estimated the prevalence of BVDV active infection in dairy herds in southern Chile to be 77% [15]. This is consistent with the model's outcome for dairy herds (Figure 9A), demonstrating that the model approach can produce results that align with real situations. Unfortunately, the overall prevalence of BVD in all herd classes in southern Chile is very outdated and cannot be compared. The available information only relates to seroprevalence in other regions of the country. However, studies show evidence of a widespread situation of the virus [16, 50]. This is also reflected in model outcomes, where the prevalence in an endemic state is nearly 64% (Figure 7A).

#### 4.5.5. Sensitivity Analysis for Uncertainty Sources

The sensitivity analysis focused on factors that influence transmission among herds and can be a source of considerable uncertainty. In the model, BVDV transmission is triggered only by movements. Therefore, prevalence and movement frequency are crucial elements for transmission probability values. The prevalence values used in the model were from dairy herds and extrapolated to the other herd classes due to the lack of information on BVDV prevalence in those classes, which may introduce uncertainty. Modifying transmission probabilities affects both the overall prevalence and the time to reach an endemic state, highlighting the importance of these factors.

Cattle movement plays a critical factor in the BVDV spread [9, 11]; in the absence of movement, for example, the infection does not persist, as reported by Courcoul and Ezanno [24]. The use of existing shipment data and actual prevalence information for dairy herds in southern Chile helps to control uncertainty caused by listed limitations to some extent and adds realism to the simulation [51]. Neighboring transmission is not considered in this approach, as it is unlikely due to the extensive production systems used in southern Chile. This reduces the probability of effective contact by neighboring, such as common pasture use or contact through fences.

## 5. Conclusions

Specialized software can be used to model complex livestock disorders like BVDV by integrating within and between-herd dynamics.

The representation of herd infection dynamics into six profiles enables their use as an input for herd state in ISP, allowing for simulation of transitions between them. The presented approach successfully modeled the complex infection dynamics of BVDV with confidence, suggesting its potential for application to other complex infections where internal dynamics significantly impact pathogen spread in metapopulations.

The sensitivity analysis of BHM parameters indicates that the transmission probability between herds is a critical factor for model outcomes. Reliable information on transmission probability for the remaining herd categories would be helpful in reducing uncertainty in the results.

Future research using this model will explore mitigation strategies to control BVDV in southern Chilean herds, and it could be extended to investigate other infectious disease control strategies.

## Figures and Tables

**Table 1:** WHM parameters.

Parameter	Definition	Value	Source
*Br*	Birth rate^a^	0.0027	One calf per cow/year assumed
*Cr*	Calves culling rate^a^ (per day)	0.00027	Derived from [32]
*Rr*	General replacement rate^a^ (per day)	0.00054	Derived from [32]
*β*_*g*_^*T*^	Transmission rate for TI animals (per day)	0.002	[34]
*β*_*g*_^*P*^	Transmission rate for PI animals (per day)	0.03	[34]
*β*_*b*_^*Pi*^	Between-group transmission rate for PI animals (per day)	0.0071	[35]
*Rcr*	Recovery rate (per day)	0.071	[36]
*m*_ti_	TI mortality (per day)	0.017	[37]
*m*_pi_	PI mortality probability (per day)	0.0012	[38]
*T*_1a_	Reciprocal of the duration time of maternal antibody protection (1/180 days)	0.006	[34]
*T*_1b_	Reciprocal of the time spent as Sus calve after losing maternal protection to Sus heifer (1/220 days)	0.0045	Derived [32]
*T*_2_	Reciprocal of the time spent as Sus calve without maternal protection at birth to Sus heifer (1/400 days)	0.0025	[32]
*T*_3_	Reciprocal of the time spent in every pregnancy trimester (1/90 days)	0.01	—
CPI	Calving to pregnancy interval (1/90)	0.011	[33]

Abbreviations: PI, persistently infected; Sus, susceptible; TI, transiently infected.

^a^Parameters for Chilean dairy herds.

**Table 2:** Probabilities of different outcomes after infection in pregnant heifers and cows.

Outcome	Trimester		
	1st	2nd	3rd
PI calf	0.60	0.14	0.001
Fetal-infected calf	0.29	0.53	0.946
Aborted calf	0.11	0.04	0.053
Congenital defects calf	—	0.29	—

*Note*: Extracted from [40].

Abbreviation: PI, persistently infected.

**Table 3:** Herd-profiles transition probabilities matrix for small dairy herds.

Herd-profile	Infected low immunity	Infected medium immunity	Infected high immunity	Clear low immunity	Clear medium immunity	Clear high immunity
Infected low immunity	0.05	0.86	0.01	0.06	0.02	—
Infected medium immunity	—	0.35	0.52	—	0.09	0.04
Infected high immunity	—	0.01	0.90	—	0.02	0.07
Clear low immunity	—	—	—	1	—	—
Clear medium immunity	—	—	—	0.08	0.92	—
Clear high immunity	—	—	—	—	0.49	0.51

**Table 4:** Herd-profiles transition probabilities matrix for medium dairy herds.

Herd-profile	Infected low immunity	Infected medium immunity	Infected high immunity	Clear low immunity	Clear medium immunity	Clear high immunity
Infected low immunity	0.33	0.67	—	—	—	—
Infected medium immunity	—	0.52	0.45	—	0.03	—
Infected high immunity	—	—	0.98	—	—	0.02
Clear low immunity	—	—	—	1	—	—
Clear medium immunity	—	—	—	0.03	0.97	—
Clear high immunity	—	—	—	—	0.18	0.82

**Table 5:** Herd-profiles transition probabilities matrix for large dairy herds.

Herd-profile	Infected low immunity	Infected medium immunity	Infected high immunity	Clear low immunity	Clear medium immunity	Clear high immunity
Infected low immunity	—	0.02	0.98	—	—	—
Infected medium immunity	—	0.76	0.04	—	0.20	—
Infected high immunity	—	—	0.97	—	—	0.03
Clear low immunity	—	—	—	1	—	—
Clear medium immunity	—	—	—	0.08	0.92	—
Clear high immunity	—	—	—	—	0.53	0.47

**Table 6:** Parameters values (*λ* for a Poisson distribution) to represent shipment frequency every 90 days for different herd classes and locations.

Herd class	Herd location	
	Valdivia province	Ranco province
Cow calf large	4.37	5.13
Cow calf medium	4.32	3.9
Cow calf small	3.7	3.4
Dairy large	8.42	7.5
Dairy medium	4.2	4.5
Dairy small	3.2	2.6
Fattening large	4.7	4.5
Fattening medium	4.32	3.8
Fattening small	3.7	3
Market	76.1	349
Mixed large	10.5	6.7
Mixed medium	4.26	2.7
Mixed small	3.11	2.8
Others large	3.6	1.5
Others medium	1	—
Others small	3.23	4.2
Sport/exhibition centers	4.2	4.5

*Note*: Movement data for *λ* were obtained from [43].

**Table 7:** Between-herd transmission probabilities for different herds and herd sizes.

Herd type	Herd size	Function	Parameter value
Farm	Large	*pbinom*	*n* = 19, *p* = 0.035
	Medium	*pbinom*	*n* = 12, *p* = 0.035
	Small	*pbinom*	*n* = 6, *p* = 0.035
Markets	—	*pbinom*	*n* = 6, *p* = 0.035
Sport/exhibition centers	—	*pbinom*	*n* = 19, *p* = 0.035

**Table 8:** Median number of newly infected herds caused by the different herd types.

Source of infection	Infected herd class							
	Dairy	Cow calf	Fattening	Market	Mixed	Others	Sport/exhibition center	Total
Cow calf	188	432	1216	3	149	63	12	2062
Dairy	1663	718	4616	7	787	244	36	8070
Fattening	209	—	—	1	179	29	2	420
Market	334	362	1984	2	286	109	17	3094
Mixed	556	414	1477	3	501	66	10	3027
Others	13	16	47	1	7	3	1	88
Sport/exhibition center	15	4	102	1	1	—	3	126

## Data Availability

The datasets generated during and/or analyzed in the current study are available from the corresponding author upon reasonable request.

## Supporting Information

Supporting Information 1 Destination probability of animal shipments of herds from Valdivia Province.

Supporting Information 2 Destination probability of animal shipments of herds from Ranco Province.

Supporting Information 3 WHM transition probabilities for small, medium, and large herds of mixed and fattening herd classes (Table S3 1–6).
