# Supplementary material for: Herd-Level Modeling of Bovine Viral Diarrhea Virus (BVDV) Transmission in Cattle Herds in Southern Chile: Linking Within and Between-Herd Dynamics
Source: Transbound Emerg Dis. 2024 Oct 28;2024:4734277. doi: 10.1155/2024/4734277 (PMC12017151; doi:10.1155/2024/4734277)
Supplement: Supporting Information 1 — Destination probability of animal shipments of herds from Valdivia Province. [file 4734277.f1.pdf]

**Suplementatary 1. Destination probability for herds from Valdivia province**

Destination Herd Class — Ranco

| Source Herd Class | CowCalf Large | CowCalf Medium | CowCalf Small | Fattening Large | Fattening Medium | Fattening Small | Dairy Large | Dairy Medium | Dairy Small | Mixed Large | Mixed Medium | Mixed Small | Others Small | Market |
|---|---|---|---|---|---|---|---|---|---|---|---|---|---|---|
| Valdivia CowCalf Large | 0,067 | 0,020 | 0,004 | 0,056 | 0,001 | 0,006 | 0,002 | 0,001 | 0,001 | 0,104 | | 0,004 | 0,002 | 0,007 |
| Valdivia CowCalf Medium | 0,012 | 0,002 | 0,001 | 0,010 | 0,002 | | 0,001 | | 0,001 | 0,002 | 0,001 | | | 0,015 |
| Valdivia CowCalf Small | 0,000 | | | 0,000 | 0,000 | 0,000 | | | 0,000 | 0,000 | | 0,000 | 0,000 | 0,003 |
| Valdivia Fattening Large | | | | | | | 0,066 | 0,028 | | | 0,002 | 0,002 | | 0,014 |
| Valdivia Fattening Medium | | | | | | | 0,004 | 0,011 | | | 0,004 | | | 0,018 |
| Valdivia Fattening Small | | | | | | | 0,000 | 0,001 | 0,001 | 0,002 | | | | 0,011 |
| Valdivia Dairy Large | 0,003 | 0,002 | 0,011 | 0,016 | 0,001 | 0,023 | 0,014 | 0,005 | 0,020 | 0,001 | 0,001 | 0,010 | 0,006 | 0,004 |
| Valdivia Dairy Medium | | | 0,005 | 0,002 | 0,001 | | 0,002 | 0,026 | 0,011 | | 0,001 | | 0,002 | 0,002 |
| Valdivia Dairy Small | | | 0,001 | | | 0,002 | 0,002 | | 0,003 | | 0,000 | | | 0,005 |
| Valdivia Mixed Large | 0,021 | 0,002 | 0,001 | 0,049 | | 0,003 | 0,035 | 0,009 | 0,020 | 0,006 | 0,000 | 0,002 | 0,000 | 0,005 |
| Valdivia Mixed Medium | | | 0,002 | 0,034 | | 0,002 | | | 0,002 | | | | | |
| Valdivia Mixed Small | | 0,001 | | | | 0,001 | | | 0,002 | | | 0,001 | 0,001 | 0,016 |
| Valdivia Others Large | | | | | | | | | | | | | | |
| Valdivia Others Medium | | | | | | | | | | | | | | |
| Valdivia Others Small | | | | | 0,005 | | | | | | | 0,005 | | |
| Valdivia Market | 0,013 | 0,010 | 0,018 | 0,037 | 0,012 | 0,030 | 0,002 | 0,004 | 0,034 | 0,001 | | 0,022 | 0,007 | |
| Valdivia Sport-Exhibition Center | | | | 0,095 | | | | | | | | | | |

Destination Herd Class — Valdivia

| Source Herd Class | CowCalf Large | CowCalf Medium | CowCalf Small | Fattening Large | Fattening Medium | Fattening Small | Dairy Large | Dairy Medium | Dairy Small | Mixed Large | Mixed Medium | Mixed Small | Others Large | Others Medium | Others Small | Market | Sport-Exhibition Center |
|---|---|---|---|---|---|---|---|---|---|---|---|---|---|---|---|---|---|
| Valdivia CowCalf Large | 0,066 | 0,018 | 0,011 | 0,082 | 0,063 | 0,068 | 0,053 | 0,080 | 0,004 | 0,046 | 0,007 | 0,004 | 0,001 | 0,006 | | 0,190 | 0,028 |
| Valdivia CowCalf Medium | 0,010 | 0,010 | 0,011 | 0,008 | 0,015 | 0,024 | 0,002 | 0,003 | 0,011 | 0,006 | 0,004 | 0,005 | | | 0,002 | 0,839 | 0,004 |
| Valdivia CowCalf Small | | 0,005 | 0,012 | 0,005 | 0,004 | 0,018 | | | 0,005 | 0,001 | | 0,003 | | | 0,001 | 0,940 | 0,001 |
| Valdivia Fattening Large | | | | | | | 0,021 | 0,002 | 0,024 | 0,019 | 0,017 | 0,005 | | | | 0,767 | 0,033 |
| Valdivia Fattening Medium | | | | | | | 0,083 | 0,061 | 0,076 | 0,032 | 0,083 | 0,047 | | | 0,022 | 0,538 | 0,022 |
| Valdivia Fattening Small | | | | | | | 0,005 | 0,008 | 0,011 | 0,044 | 0,007 | 0,001 | | | | 0,905 | 0,006 |
| Valdivia Dairy Large | 0,005 | 0,013 | 0,023 | 0,023 | 0,037 | 0,124 | 0,177 | 0,023 | 0,062 | 0,200 | 0,010 | 0,021 | 0,001 | | 0,015 | 0,147 | 0,004 |
| Valdivia Dairy Medium | 0,090 | 0,002 | 0,018 | 0,061 | 0,032 | 0,056 | 0,074 | 0,023 | 0,020 | 0,086 | 0,028 | 0,011 | | | 0,002 | 0,442 | 0,001 |
| Valdivia Dairy Small | 0,000 | 0,003 | 0,013 | 0,005 | 0,002 | 0,023 | 0,032 | 0,006 | 0,040 | 0,012 | 0,002 | 0,005 | | | 0,001 | 0,841 | 0,001 |
| Valdivia Mixed Large | 0,034 | 0,007 | 0,023 | 0,068 | 0,024 | 0,075 | 0,312 | 0,019 | 0,041 | 0,056 | 0,033 | 0,043 | 0,002 | 0,005 | 0,006 | 0,096 | 0,002 |
| Valdivia Mixed Medium | | 0,008 | 0,013 | 0,050 | 0,003 | 0,020 | 0,070 | 0,056 | 0,019 | 0,027 | 0,028 | 0,009 | | | | 0,657 | 0,000 |
| Valdivia Mixed Small | | 0,009 | 0,007 | 0,007 | 0,001 | 0,025 | 0,008 | 0,001 | 0,007 | 0,006 | 0,002 | 0,004 | | | 0,001 | 0,900 | 0,001 |
| Valdivia Others Large | | | | 0,091 | 0,455 | 0,091 | | | | | | | | | | | 0,364 |
| Valdivia Others Medium | | | 0,500 | | | | | | | | | | | | | 0,500 | 0,000 |
| Valdivia Others Small | | 0,016 | 0,005 | | | 0,005 | | | 0,010 | | | | | | | 0,817 | 0,136 |
| Valdivia Market | 0,002 | 0,083 | 0,112 | 0,191 | 0,034 | 0,266 | 0,001 | 0,001 | 0,049 | 0,003 | 0,017 | 0,040 | | | 0,007 | 0,003 | 0,000 |
| Valdivia Sport-Exhibition Center | | | | 0,429 | 0,048 | | 0,190 | | | | | | | | | 0,048 | 0,190 |
