# Supplementary material for: Herd-Level Modeling of Bovine Viral Diarrhea Virus (BVDV) Transmission in Cattle Herds in Southern Chile: Linking Within and Between-Herd Dynamics
Source: Transbound Emerg Dis. 2024 Oct 28;2024:4734277. doi: 10.1155/2024/4734277 (PMC12017151; doi:10.1155/2024/4734277)
Supplement: Supporting Information 2 — Destination probability of animal shipments of herds from Ranco Province. [file 4734277.f2.pdf]

Suplementary 2. Destination probability for herds from Ranco province

Destination Herd Class

Ranco

Source Herd Class	CowCalf Large	CowCalf Medium	CowCalf Small	Fattening Large	Fattening Medium	Fattening Small	Dairy Large	Dairy Medium	Dairy Small	Mixed Large	Mixed Medium	Mixed Small	Others Large	Others Small	Market	Sport-Exhibition center
Ranco CowCalf Large	0,263	0,094	0,014	0,044	0,026	0,030	0,008	0,021	0,014	0,018	0,006	0,006		0,026	0,161	0,019
Ranco CowCalf Medium	0,051	0,003	0,002	0,010	0,002	0,017	0,025		0,014	0,011	0,006			0,003	0,240	0,001
Ranco CowCalf Small	0,004	0,003	0,009	0,001	0,002	0,042	0,001	0,001	0,012		0,001	0,015	0,001	0,007	0,419	0,001
Ranco Fattening Large							0,035	0,006	0,003	0,001	0,004	0,018		0,003	0,280	0,003
Ranco Fattening Medium							0,023	0,034	0,004	0,011	0,041	0,019	0,004	0,006	0,305	
Ranco Fattening Small							0,002	0,004	0,004	0,012	0,005	0,003		0,001	0,411	0,001
Ranco Dairy Large	0,018	0,029	0,029	0,040	0,026	0,130	0,170	0,048	0,074	0,043	0,007	0,025	0,004	0,032	0,116	
Ranco Dairy Medium	0,012	0,005	0,006	0,010	0,016	0,053	0,184	0,082	0,031	0,018	0,010	0,015		0,012	0,283	
Ranco Dairy Small	0,004	0,007	0,009	0,002	0,016	0,026	0,014	0,014	0,032		0,001	0,014	0,004	0,009	0,476	
Ranco Mixed Large	0,157	0,028	0,053	0,057	0,050	0,053	0,075	0,031	0,038	0,012	0,023	0,037	0,003	0,013	0,181	0,002
Ranco Mixed Medium	0,032	0,007	0,005	0,007	0,032	0,018	0,050	0,038	0,016	0,063	0,007	0,029	0,002	0,007	0,317	0,002
Ranco Mixed Small	0,002	0,009	0,005		0,001	0,020	0,018	0,003	0,008	0,003	0,002	0,015		0,003	0,475	
Ranco Others Large					0,125									0,375	0,250	
Ranco Others Small	0,005		0,005	0,002	0,013	0,009	0,008		0,013			0,007	0,002		0,590	
Ranco Market	0,056	0,035	0,065	0,070	0,039	0,321	0,028	0,029	0,136	0,002	0,011	0,093	0,012	0,074	0,001	
Ranco Sport-Exhibition Center	0,667														0,333	

Valdivia

Source Herd Class	CowCalf Large	CowCalf Medium	CowCalf Small	Fattening Large	Fattening Medium	Fattening Small	Dairy Large	Dairy Medium	Dairy Small	Mixed Large	Mixed Medium	Mixed Small	Others Small	Market	Sport-Exhibition Center
Ranco CowCalf Large	0,053	0,036	0,001	0,024		0,005	0,009		0,001	0,034	0,001		0,001	0,081	0,004
Ranco CowCalf Medium	0,003		0,001	0,003	0,003	0,002				0,009				0,593	
Ranco CowCalf Small			0,008			0,017		0,001	0,010		0,002	0,001		0,439	0,003
Ranco Fattening Large							0,031	0,060	0,015	0,001	0,001	0,009	0,012	0,515	0,001
Ranco Fattening Medium								0,004						0,551	
Ranco Fattening Small							0,000	0,000	0,001	0,003	0,001	0,000	0,000	0,549	0,002
Ranco Dairy Large	0,001	0,003	0,013	0,007	0,000	0,031	0,019	0,002	0,025	0,008	0,003	0,008	0,001	0,087	0,002
Ranco Dairy Medium	0,002		0,001		0,002	0,001	0,022	0,013		0,014	0,001			0,208	0,001
Ranco Dairy Small			0,002	0,003		0,008	0,001	0,000	0,003	0,000	0,000	0,004		0,352	
Ranco Mixed Large	0,039	0,001	0,002	0,014	0,001	0,004	0,014	0,000		0,010	0,001	0,001	0,002	0,093	0,002
Ranco Mixed Medium						0,002								0,367	
Ranco Mixed Small				0,002		0,002						0,001		0,430	
Ranco Others Large														0,250	
Ranco Others Small			0,001	0,005		0,002								0,338	0,001
Ranco Market		0,002		0,004		0,004							0,001		0,015
Ranco Sport-Exhibition Center															
