# Supplementary material for: Herd-Level Modeling of Bovine Viral Diarrhea Virus (BVDV) Transmission in Cattle Herds in Southern Chile: Linking Within and Between-Herd Dynamics
Source: Transbound Emerg Dis. 2024 Oct 28;2024:4734277. doi: 10.1155/2024/4734277 (PMC12017151; doi:10.1155/2024/4734277)
Supplement: Supporting Information 3 — WHM transition probabilities for small, medium, and large herds of mixed and fattening herd classes (Table S3 1–6). [file 4734277.f3.pdf]

S3 Table 1. Small Mixed herd-profiles transition probabilities.

	Infected Low Immunity	Infected Medium Immunity	Infected High Immunity	Clear Low Immunity	Clear Medium Immunity	Clear High Immunity
**Infected Low Immunity**	0,01	0,86	0,10	0,02	0,02	
**Infected Medium Immunity**		0,16	0,70		0,11	0,03
**Infected High Immunity**			0,93			0,07
**Clear Low Immunity**				1,00		
**Clear Medium Immunity**				0,08	0,92	
**Clear High Immunity**					0,53	0,47

S3 Table 2. Medium Mixed herd-profiles transition probabilities.

	Infected Low Immunity	Infected Medium Immunity	Infected High Immunity	Clear Low Immunity	Clear Medium Immunity	Clear High Immunity
**Infected Low Immunity**	0,01	0,92	0,01	0,04	0,01	
**Infected Medium Immunity**		0,42	0,46		0,11	0,01
**Infected High Immunity**			0,94		0,01	0,06
**Clear Low Immunity**				1,00		
**Clear Medium Immunity**				0,09	0,91	
**Clear High Immunity**					0,67	0,33

S3 Table 3. Large Mixed herd-profiles transition probabilities.

	Infected Low Immunity	Infected Medium Immunity	Infected High Immunity	Clear Low Immunity	Clear Medium Immunity	Clear High Immunity
**Infected Low Immunity**	0,02	0,89	0,04	0,04	0,01	
**Infected Medium Immunity**		0,31	0,59		0,09	0,01
**Infected High Immunity**			0,97			0,03
**Clear Low Immunity**				1,00		
**Clear Medium Immunity**				0,09	0,91	
**Clear High Immunity**					0,69	0,31

S3 Table 4. Small Fattening herd-profiles transition probabilities.

	Infected Low Immunity	Infected Medium Immunity	Infected High Immunity	Clear Low Immunity	Clear Medium Immunity	Clear High Immunity
**Infected Low Immunity**		0,03	0,72	0,01	0,05	0,19
**Infected Medium Immunity**		0,13			0,38	0,50
**Infected High Immunity**		0,01	0,59			0,41
**Clear Low Immunity**				0,81	0,13	0,06
**Clear Medium Immunity**					0,29	0,71
**Clear High Immunity**						1,00

S3 Table 5. Medium Fattening herd-profiles transition probabilities.

	**Infected Low Immunity**	**Infected Medium Immunity**	**Infected High Immunity**	**Clear Low Immunity**	**Clear Medium Immunity**	**Clear High Immunity**
**Infected Low Immunity**			0,75			0,25
**Infected Medium Immunity**						
**Infected High Immunity**			0,71			0,29
**Clear Low Immunity**				1,00		
**Clear Medium Immunity**						
**Clear High Immunity**						1,00

S3 Table 6. Large Fattening herd-profiles transition probabilities.

	**Infected Low Immunity**	**Infected Medium Immunity**	**Infected High Immunity**	**Clear Low Immunity**	**Clear Medium Immunity**	**Clear High Immunity**
**Infected Low Immunity**			0,73			0,27
**Infected Medium Immunity**						
**Infected High Immunity**			0,65			0,35
**Clear Low Immunity**						
**Clear Medium Immunity**						
**Clear High Immunity**						1,00
